# Neonatal and infant mortality associated with spina bifida: A systematic review and meta-analysis

**DOI:** 10.1371/journal.pone.0250098

**Published:** 2021-05-12

**Authors:** Peter Ho, Maria A. Quigley, Dharamveer Tatwavedi, Carl Britto, Jennifer J. Kurinczuk

**Affiliations:** 1 National Perinatal Epidemiology Unit (NPEU), Nuffield Department of Population Health, University of Oxford, Oxford, United Kingdom; 2 Radcliffe Department of Medicine, University of Oxford, Oxford, United Kingdom; 3 Oxford Vaccine Group, University of Oxford, Oxford, United Kingdom; Center of Pediatrics, GERMANY

## Abstract

**Objectives:**

A systematic review was conducted in high-income country settings to analyse: (i) spina bifida neonatal and IMRs over time, and (ii) clinical and socio-demographic factors associated with mortality in the first year after birth in infants affected by spina bifida.

**Data sources:**

PubMed, Embase, Ovid, Web of Science, CINAHL, Scopus and the Cochrane Library were searched from 1^st^ January, 1990 to 31^st^ August, 2020 to review evidence.

**Study selection:**

Population-based studies that provided data for spina bifida infant mortality and case fatality according to clinical and socio-demographical characteristics were included. Studies were excluded if they were conducted solely in tertiary centres. Spina bifida occulta or syndromal spina bifida were excluded where possible.

**Data extraction and synthesis:**

Independent reviewers extracted data and assessed their quality using MOOSE guideline. Pooled mortality estimates were calculated using random-effects (+/- fixed effects) models meta-analyses. Heterogeneity between studies was assessed using the Cochrane Q test and I^2^ statistics. Meta-regression was performed to examine the impact of year of birth cohort on spina bifida infant mortality.

**Results:**

Twenty studies met the full inclusion criteria with a total study population of over 30 million liveborn infants and approximately 12,000 spina bifida-affected infants. Significant declines in spina bifida associated infant and neonatal mortality rates (e.g. 4.76% decrease in IMR per 100, 000 live births per year) and case fatality (e.g. 2.70% decrease in infant case fatality per year) were consistently observed over time. Preterm birth (RR 4.45; 2.30–8.60) and low birthweight (RR 4.77; 2.67–8.55) are the strongest risk factors associated with increased spina bifida infant case fatality.

**Significance:**

Significant declines in spina bifida associated infant/neonatal mortality and case fatality were consistently observed, advances in treatment and mandatory folic acid food fortification both likely play an important role. Particular attention is warranted from clinicians caring for preterm and low birthweight babies affected by spina bifida.

## Introduction

Neural tube defects (NTDs) constitute the largest group of congenital anomalies of the central nervous system [1]; the aetiology of spina bifida is multifactorial [2,3]. Mortality among infants with spina bifida has been previously investigated in several studies, mainly restricted to selected geographical regions especially in high-income countries where data are more available [4].

Infant mortality associated with spina bifida has been changing over time depending on various factors including folic acid supplementation and food fortification programme [5], prenatal screening [6], treatment and termination of pregnancy [7], and the health care system to tract and link all cases with death registers [4]. We conducted a systematic review and meta-analysis of population-based studies focusing on liveborn infants with spina bifida in high-income countries.

The aims of this study were to assess: (1) spina bifida- specific neonatal and infant mortality rates over time; and (2) the socio-demographic and clinical factors associated with mortality in the first year for infants affected by spina bifida.

## Methods

The methods for the overall systematic review have been registered as a review protocol in the International Prospective Register of Systematic Reviews (PROSPERO) [8], registration number CRD42018081353.

### Search strategy

Comprehensive literature searches of PubMed, Embase, Ovid, Web of Science, CINAHL, Scopus and the Cochrane Library was performed from 1^st^ January, 1990 to 31^st^ August, 2020. MeSH terms and keywords which included *neural tube defect* or *congenital brain malformation* or *abnormalities* or *spina bifida* and *infant* or *neonatal or perinatal* and *mortality* or *death* or *survival* etc. were entered systematically into the databases. The detailed search terms are shown in S1 Table. There were no language restrictions applied. Manual searches of reference lists were performed on all the included publications. Attempts were also made to contact the study authors for any relevant unpublished data where appropriate.

Titles and abstracts of all identified studies were screened by PH according to the inclusion criteria. For articles which satisfied the first screen, full articles were then screened independently by two reviewers (PH and DT/ CB) for their eligibility according to the full inclusion and exclusion criteria. Any discrepancies were reviewed by the author review team (PH, MQ and JK) to reach a final decision.

### Eligibility criteria

Population-based studies were included if they (1) ascertained all individuals born with spina bifida in a predefined population; (2) provided data for spina bifida infant mortality (defined as the number of spina bifida associated deaths under one year of age occurring among all livebirths in a given population) or case fatality (defined as the proportion of spina bifida associated deaths among all spina bifida cases under one year of age in a given population), or case fatality estimates according to clinical or socio-demographical characteristics; and (3) were conducted in high-income countries as defined by the World Bank [9].

Studies were excluded if: (1) they were conducted solely in tertiary or referral centres; and (2) spina bifida-affected individuals were not followed up from birth. Spina bifida occulta (i.e. a mild form of spina bifida with no clinical consequence) or spina bifida related to a syndrome were excluded where possible.

For multiple papers which overlapped in time, study region and objectives [3,10–22], only the study (or a combination of studies) with the best quality, largest population size, and covering the longest and most recent periods were included.

### Data extraction

PH conducted the literature searches with the assistance of a specialist information librarian, screening of abstracts and review of 368 eligible full articles. DT and CB each reviewed 50% of the eligible full articles, validated decisions about the final included articles and extracted data.

The following data were extracted: study population, study design, sample size, country of study, and descriptive/ mortality data about maternal age, maternal ethnicity, smoking, maternal education, previous live births, prenatal care, induction of labour, mode of delivery, infant sex, gestational age, birthweight, year of birth, plurality, lesion level, the presence of multiple defects, hydrocephalus and major cardiac defects and period of folic acid fortification.

Spina bifida mortality rates, case fatality and the corresponding 95% CI were extracted at the age of one month and one year. If the 95% CI of the mortality rates were not reported, they were estimated using ‘binomial exact’, assuming no cases were censored. In studies where 95% CI of relative risks (RR) of factors for mortality were not reported, they were estimated using log RR ± 1.96 × Standard Error, where standard error was derived from the counts and proportions.

#### Statistical analysis

As all the pooled estimates for mortality risk factors rate ratios came from two studies [10–12,23], both random-effects and fixed effects models were used to calculate and compare the pooled estimates. These studies were conducted during pre- mandatory folic acid food fortification period, which justified the decision for the effects pooling. Heterogeneity between studies was assessed using the Cochrane Q test and I^2^ statistics, p < 0.10 was considered as statistically significant with I^2^ > 50% indicating a substantial level of heterogeneity. Random-effects meta-regression was used to assess the effect of year of birth. Results of meta-regressions are presented in bubble plots, where the weights used to determine the bubble size are the inverses of the effect-size variances and hence the size of the bubble is proportional to the precision of each study. The slopes of the meta-regression lines were calculated, with p < 0.05 considered as statistically significant.

All data analyses were performed using Stata version 13 (StataCorp). The reporting of results was in accordance with the Preferred Reporting Items for Systematic Reviews and Meta-Analyses (PRISMA) flow diagram and Meta-analysis of Observation Studies in Epidemiology (MOOSE) checklist [24,25].

#### Quality appraisal

Risk of bias (RoB) assessment was conducted using a refined quality tool based on Quality in Prognostic Studies (QUIP) [26] for the following domains: study participation, measurement of outcome(s), study attrition, measurement of exposure or prognostic factor(s) and statistical analysis and reporting. Each domain was rated either as ‘high’, ‘moderate’ or ‘low’ RoB by two independent reviewers (PH and DT/CB). The overall RoB for each included study was assessed using the method described by Hayden et al. [27] in line with the Cochrane Risk of Bias tool [28].

## Results

**Fig 1:**
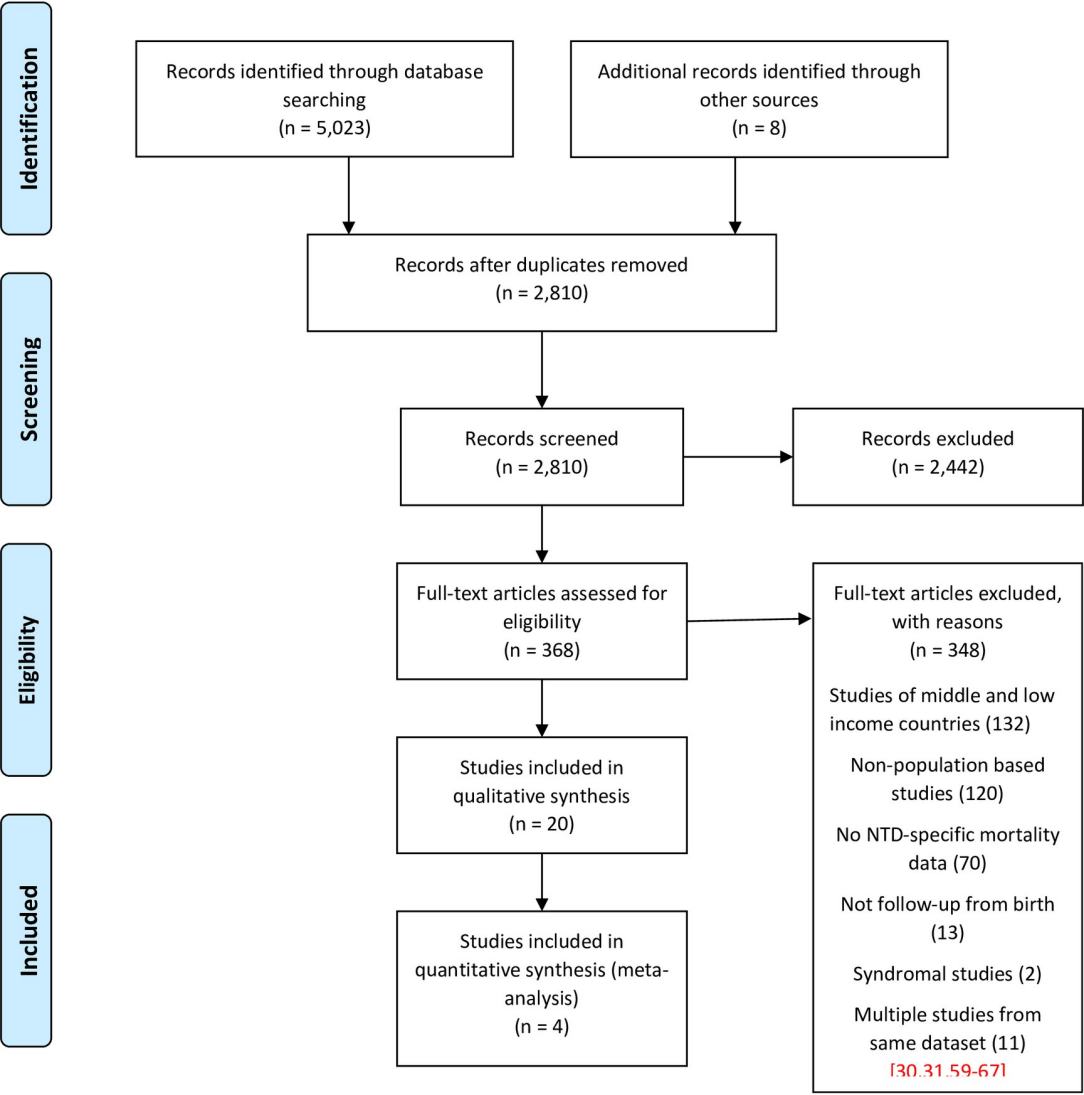
PRISMA diagram for the flow of articles through the review (1.1.1990 to 31.8.2020).

The flow of literature in this review is summarised in the PRISMA diagram (Fig 1). Of 5,023 articles identified, 20 met the inclusion criteria.

### Study characteristics

All 20 included studies were population-based cohort studies conducted in high-income countries, with six studies in the U.S. (mandatory folic acid food fortification introduced in 1998) [3,10–12,29,30], four in Canada (mandatory folic acid food fortification introduced in 1998) [31–34], four in Australia (mandatory folic acid food fortification introduced in 2009) [23,35–37], two in Republic of Ireland [38,39], one in the UK (Scotland) [40] and one in Sweden [41], one in Denmark [42], and one from the International Clearinghouse for Birth Defects Surveillance and Research (ICBDSR) selected member registries of France, Germany, Italy, Netherlands, Spain, Sweden and UK Wales [43] (Table 1). These studies included a total study population of over 30 million liveborn infants (median study sample size: 1,178,452, range: 251,699–14 million) in which approximately 12,000 (median: 231 and range: 27–3,903) were affected by spina bifida. All infants included were born at ≥20 weeks gestational age with a birthweight ≥500g.

**Table 1:** Description of the included studies.

Author, Included birth years	Study location and no. of liveborn spina bifida	Included NTD subtypes (ICD codes)	Source of cases	Source of death information	Percentage of traced cases	Inclusion of multiple defects	Main limitations
Kalucy (1994) [23] 1966–1990	Western Australia n = 395	Spina bifida ± Hydrocephalus (Coding not specified)	Birth defects registry, inpatient records.	Death certificates, post mortem reports, matched with infants’ demographics.	Not stated.	Yes; multiple and chromosomal defects were included but no further details.	Mortality status was only known for a proportion of cases.
Laishram (1993) [31] 1967–1990	Newfoundland and Labrador, Canada. n = 273	Spina bifida (Coding not specified)	Inpatient records	Death certificate (assumed)	99.6%	Yes; cardiac, gastrointestinal, urological and limbs/skeletal defects, and orofacial cleft. But only those needed operations were reported.	Diagnostic code for classification of disease not described.
Sutton (2008) [38] 1976–1987	Dublin, Ireland n = 475	Spina bifida (Coding not specified)	Four Dublin maternity hospitals medical records.	Medical record and/or parental interview.	92%	Eight cases of encephalocele had an additional spinal lesion.	Diagnostic coding for disease classification not described.
Waitzman (1994) [29] 1983–1986	California, USA n = 226	Spina bifida (ICD-9/BPA^2^: 741, 741.0x, 741.9x)	California Birth Defects Monitoring Programme.	Linked birth and death records.	Not stated.	Yes; but no further details reported.	Confidence interval of estimates not provided.
Wong (2001) [10] 1979–1994	Atlanta, USA n = 235	Spina bifida (ICD-9: 741)	Metropolitan Atlanta Congenital Defects Programme.	National Death Index and death certificates linked with registry data.	100%	Yes; but cases with anencephaly, and Trisomies 13 or 18 were excluded.	Sizes of birth cohorts limited the statistical power to detect their significance in the trend in one-year survival.
Wen (2000) [34] 1981–83, 1993–95	Canada n = Unspecified	Spina bifida (ICD-9: 740)	Nine Canadian provinces^1^, recorded in Statistics Canada’s live birth and death databases.	Statistics Canada’s livebirth and death databases.	~100%	Not stated.	As described in the study of Wen at al. (1999) [32].
Riley (1998) [35] 1983–1995	Victoria, Australia n = 526	Spina bifida (BPA ICD-9 supplement)	Victorian Birth Defects/ Congenital Malformations Register.	Perinatal death certificates, autopsy reports, maternal and child health nurse notifications, inpatient and outpatient listings; record linkage using Perinatal Morbidity Statistics System.	Up to 86%.	Not stated.	Voluntary notification to congenital malformations register, completeness depended on type of malformation.
Liu (2001) [33] 1985–1996	Canada n = unspecified	Spina bifida (ICD-9: 741)	Canadian stillbirth and infant death registration.	Statistics Canada’s Mortality Database.	98%	Not stated.	1. Death certificates recorded a single underlying cause of death. 2. Potential errors of assigning and coding the cause of death from death certificates (e.g. minor anomalies being coded as an underlying cause of death).
Borgstedt-Bakke (2017) [42] 1970–2015	Western Demark n = 187	Spina bifida (Coding not specified)	Western Denmark myelo-meningocele database.	Hospital’s record linkage (Danish Civil Registration System).	92%	Not stated.	1. Missing data likely underestimate death rate. 2. Unspecified diagnostic code for myelomeningocele.
Wen (1999) [32] 1990–1995	Canada n = unspecified	Spina bifida (ICD-9: 741)	Nine Canadian provinces, recorded in Statistics Canada’s live birth and death databases.	Statistics Canada’s livebirth and death databases.	~100%	Not stated.	1. Assumed only lethal anomalies would have been coded as the underlying causes of death. 2. Not possible to assess impact of potential regional differences in maternal exposure and primary prevention. 3. Potential errors of assigning and coding the cause of death from death certificates
Persson (2005) [41] 1989–1998	Western Sweden n = 84	Myelomeningo-coele with hydrocephalus (ICD-9 and -10)	Hospital registers in the region.	Swedish Medical Birth Registration and Statistics of the National Board of Health and Welfare.	Not stated.	The study cohort had hydro-cephalus analysed separately.	Case completeness based on assumption that all cases were referred.
Davidoff (2002) [3] 1996–1998	USA n = unspecified	Spina bifida (ICD-9: 741.0)	National Centre for Health Statistics.	Period linked birth/infant death data.	Not stated.	Not stated.	Birth certificates can be low in sensitivity in detecting birth defects. Possible miscoding errors.
Bol (2006) [11] 1995–2001	USA n = 2,841	Spina bifida (ICD-9-CM: 741.0)	Birth defects monitoring programmes from 16 states^3^.	Death certificates and record linkage with anomaly cases.	95% - 100%	Mixed. 41 (6%) cases with concurrent spina bifida and encephalo-cele were included in both cohorts.	1. Unequal time study periods with different birth defects monitoring programmes. 2. Some participating programmes were unable to submit cases for all birth years. 3. Unequal access to treatment (such as fetal surgery) between participating states. 4. Not differentiate between syndromic and non-syndromic NTDs. Accuracy of diagnosis was not verified by Colorado state.
Theodorou (2013) [40] 1990–2009	Southeast Scotland, UK n = 43	Spina bifida (ICD coded)	Audit of congenital anomalies in South East Scotland.	Death certificates/ National Registry Scotland.	43%	Syndromal defects excluded.	High proportion of loss to follow up.
Shin (2012) [12] 1997–2003	USA n = 2,259	Spina bifida (ICD-9-CM: 741.0 and 741.9)	Birth defect monitoring programmes from 10 states^4^.	Vital and medical records and the National Death Index, linked with anomaly cases.	Not stated.	2.7–12.7% of spina bifida cases had congenital heart defects, according to different states.	1. High proportions of missing data in lesion level (41%) and maternal education. 2. Regional and periodic variations of maternal and infant characteristics and variation in diagnostic coding.
Bakkar (2019) [43] 2001–2002	ICBDSR^7^ selected registry members (France, Germany, Italy, Netherlands, Spain, Sweden, Wales). n = 518	Spina bifida (ICD-9: 741 and ICD-10: Q05)	ICBDSR multi-registries.	Linkage to administrative database and death certificates/ records or other health care databases.	High (used multiple data sources).	14.6–43.6% of spina bifida cases were either part of multiple anomalies or a syndrome.	Heterogeneous methods in data contributions. Data linked to death certificates were not uniform across programmes.
Algert (2008) [36] 2001–2003	New South Wales, Australia n = 27	Spina bifida with hydrocephalus (ICD-10)	NSW Admitted Patient Data Collection.	Australian Bureau of Statistics mortality data and record linkage.	Not stated.	Yes; the study cohort had hydrocephalus analysed separately.	1. Infants diagnosed >28 days after birth were excluded. 2. Potential diagnostic coding errors. 3. Small sample size and low study power.
Wang (2015) [30] 1999–2007	USA n = 3,903	Spina bifida (ICD-9-CM / CDC^5^/ BPA^f^)	Birth defects surveillance programmes from 12 states^6^	Death certificates and National Death Index. Anomaly cases were linked to vital records.	Not stated.	Yes; but no further details reported.	1. Total number of live birth was approximate. 2. Potentially incomplete ascertainment of deaths from missed matches of study cohort to death certificates. 3. Passive cases ascertainment
Schneuer (2019) [37] 2004–2009	New South Wales, Australia n = 56	Spina bifida (ICD-10)	New South Wales Register of Congenital Conditions (RoCC).	Record linkage to New South Wales Perinatal Data Collection, and the Registry of Births, Deaths and Marriages death registration.	High (used multiple data sources).	Yes; but no further details reported.	Unknown loss to follow up due to migration.
McDonnell (2015) [39] 2009–2011	East and Southeast of the Republic of Ireland n = 89	Spina bifida (ICD-9)	EUROCAT regional congenital anomaly registers in east, south and southeast of Ireland, all maternity hospital nationally and paediatric hospitals.	Death registrations, post-mortem reports, hospital reports, inpatient enquiry system and National Perinatal Reporting System.	Not stated.	Yes; 11% (n = 21) of the liveborn and stillborn NTD cases had ≥ 1 additional major or minor anomaly. These included T13 or 18, abdominal wall defects and diaphragmatic hernia.	24% of births from women born outside Ireland, who might have different risk profile for NTD compared to Irish-born mothers.

^1^Wen (2000) [34]: Data from the province of Newfoundland, British Columbia and Ontario were excluded in the original study.

^2^BPA = British Paediatric Association Classification of Diseases codes.

^3^Bol (2006) [11]: 16 states/ regions included Alabama, California, Colorado, Hawaii, Illinois, Iowa, Kentucky, metropolitan Atlanta, Michigan, New York, North Carolina, Oklahoma, Rhode Island, South Carolina, Texas, and West Virginia.

^4^Shin (2012) [12]: 10 regions included Arkansas, Georgia (5 counties of metropolitan Atlanta), California, Colorado, Iowa, North Carolina, New York (excluding New York City), Oklahoma, Texas and Utah.

^5^CDC = Centres for Disease Control and Prevention.

^6^Wang (2015) [30]: 12 states included Arizona, Colorado, Florida, Georgia (five counties of metropolitan Atlanta), Illinois, Massachusetts, Michigan, Nebraska, New Jersey, New York (excluded New York City), North Carolina, and Texas.

^7^ICBDSR = International Clearinghouse for Birth Defects Surveillance and Research.

#### Multiple defects

One study [41] reported that 37% of spina bifida cases were associated with hydrocephalus which was analysed separately. Eleven studies [29,10–12,24,31,38–40,43] reported that between 2.7% and 43.6% of spina bifida cases were associated with multiple anomalies or a syndrome. One study [44] reported that 7% of spina bifida cases were associated with major cardiac defects.

Cases of syndromal spina bifida were excluded from the analysis in three studies [10,39,40]; Whether syndromal spina bifida were included in other studies [3,10–12,23,29–36,38,41] was uncertain as we were unable to verify this with the authors.

### Mortality estimates

Decreasing trends in neonatal and infant mortality rates, and case fatality from spina bifida were observed in different high-income countries over time (Tables 2 and 3). For example, the spina bifida neonatal mortality rate (NMR) was 17.0 (95% CI: 14.2–19.8) per 100, 000 live births in 1983–1995 in Australia during pre- mandatory folic acid food fortification [35], compared to the spina bifida NNR at 1.6 (95% CI: 1.4–1.8) per 100, 000 live births in 1999–2007 [30] in the U.S. during post- mandatory folic acid fortification. In Canada and the U.S., the spina bifida infant IMR reduced from 23.0 (95% CI: 19.3–27.2) per 100, 000 live births in 1981–1983 [34] to 2.3 (95% CI: 2.0–2.5) per 100, 000 live births in 1990–2007 [30]. Similarly, in Australia, the spina bifida neonatal case fatality decreased from the highest reported 63% (95% CI: 55–72%) in 1966–1972 [23] to the lowest reported 16% (95% CI: 7–24%) in 1986–1990 [23]; and the spina bifida infant case fatality decreased from the highest at 68.3% in 1973–79 to the lowest at 19.6% in 2004–09 (pre-mandatory folic acid food fortification) [23,37]. In Canada, the infant case fatality decreased from 31% (95% CI: 23–40%) in 1967–1974 [31] to 9% (95% CI: 5–13%) in 1975–1990 [31].

**Table 2:** Neonatal and infant mortality rates from spina bifida.

Studies (years of birth)	Country of study (year of introduction of mandatory folic acid fortification)	Mid-year of birth cohort	Population size (no. of livebirths)	Neonatal and infant mortality rate per 100, 000 live births *(no*. *of death) [95%CI]*	
				Neonatal	Infant
**Spina bifida**					
Wen (1981–83) [34]^a^	Canada (1998)	1982	600,000		23.0 (138) [19.3–27.2]
Riley (1983–95) [35]	Victoria, Australia (2009)	1989	825,051	17.0 (140) [14.4–20.0]	
Liu (1985–87) [33]	Canada (1998)	1986	692,556		11.1 (77) [8.7–13.9]
Wen (1993–95) [34]	Canada (1998)	1994	117,8452		8.1 (96) [6.6–10.0]
Davidoff (1989–98) [3]	USA (1998)	1993/94	11,713,941		1.3 (151) [1.1–1.5]
Theodorou (1990–09) [40]	SE Scotland	1999/2000	276,404	2.2 (6) [0.8–4.7]	
Wang (1999–07) [30]	USA (1998)	2003	14,000,000	1.6 (222) [1.4–1.8]	2.3 (318) [2.0–2.5]
**Heterogeneity I**^**2**^ **and P value**				**99.6%, p = 0.000**	**99.5%, p = 0.000**
**Spina bifida with hydrocephalus**					
Persson (1989–98) [41]	Western Sweden	1993/94	253,378		2.0 (5) [0.6–4.6]
Algert (2001–03) [36]	New South Wales, Australia (2009)	2002	251,699		1.2 (3) [0.2–3.5]
**Heterogeneity I**^**2**^ **and P value**					**0.0%, p = 0.550**

^a^Data from Wen 1981–83 and 1993–95 were extracted from Wen (2000) [21].

**Table 3:** Neonatal and infant case fatality from spina bifida.

Studies (year of birth cohort)	Country of study (year of introduction of mandatory folic acid fortification)	Mid-year of birth cohort	No. of liveborn spina bifida	No. of termination	Case Fatality as % of live births affected by spina bifida *(no*. *of deaths) [95% CI]*	
					Neonatal	Infant
**Spina bifida**						
Kalucy (1966–72) [23]^a^	Australia (2009)	1969	95	0	16.8 (16) [9.3–24.4]	33.7 (32) [24.2–43.2]
Laishram (1967–74) [31]^a^	Canada (1998)	1970/71	108	Unavailable	-	31.4 (34) [22.6–40.1]
Kalucy (1973–79) [23]^a^	Australia (2009)	1976	126	0	63.5 (80) [55.1–71.9]	68.3 (86) [60.1–76.4]
Laishram (1975–90) [31]^a^	Canada (1998)	1982/83	165	Unavailable	-	9.0 (15) [4.6–13.4]
Sutton (1976–87) [38]	Republic of Ireland	1981/82	475	Unavailable	29.6 (141) [25.5–33.7]	54.9 (261) [50.5–59.4]
Wong (1979–94) [10]	USA (1998)	1986/87	235	Unavailable	9.8 (23) [5.9–13.5]	12.8 (30) [8.4–16.9]
Kalucy (1980–85) [23]^a^	Australia (2009)	1982/83	97	7	36.1 (35) [26.5–45.6]	51.5 (50) [41.6–61.5]
Riley (1983–95) [35]	Australia (2009)	1989	526	179	26.6 (140) [22.8–30.4]	-
Waitzman (1983–86) [29]	USA (1998)	1984/85	226	Unavailable	-	19.7 (44) [14.5–24.9]
Kalucy (1986–90) [23]^a^	Australia (2009)	1988	77	17	15.6 (12) [7.5–23.7]	28.6 (22) [18.5–38.7]
Borgstedt-Bakke (1970–2015) [42]	Denmark	1992/93	187	Unavailable	-	7.0 (13) [3.3–10.6]
Theodorou (1990–09) [40]	Scotland, UK	1999/2000	43	Unavailable	14.0 (6) [3.6–24.3]	-
Wang (1999–07) [30]	USA (1998)	2003	3,903	Unavailable	5.7 (222) [5.0–6.5]	8.1 (316) [7.3–9.1]
Schneuer (2004–09) [37]	Australia (2009)	2006	56	Unavailable	19.6 (11) [9.2–30]	19.6 (11) [9.2–30.0]
Bakkar (2001–12) [43]	ICBDSR registries: Paris, France Soxony Anhalt, Germany Lombardy, Italy Tuscany, Italy North, Netherlands Sweden Wales, UK	2006 2006 2007 2006 2006 2006 2006	34 41 25 22 55 263 78	149 78 38 82 41 317 216	8.7 (3) [0.0–18.4] 0 (0) [0.0–0.0] 0 (0) [0.0–0.0] 4.5 (1) [0.0–13.2] 38.2 (21) [25.3–51.0] 6.1 (16) [3.2–9.0] 9.0 (7) [2.6–15.3]	- 0 (0) [0.0–0.0] 0 (0) [0.0–0.0] 4.5 (1) [0.0–13.2] 38.2 (21) [25.3–51.0] 7.6 (20) [4.4–10.8] 9.0 (7) [2.6–15.3]
McDonnell (2009–11) [39]	Republic of Ireland	2010	89	9	5.6 (5) [0.8–10.4]	-
**Heterogeneity I**^**2**^ **and P value**					**98.7%, p < 0.001**	**99.1%, p < 0.001**
**Spina bifida with hydrocephalus**						
Kalucy (1966–90) [23]	Australia (2009)	1966	Unavailable	Unavailable	-	57.1 [50.8–63.3]
Persson (1989–98) [41]	Western Sweden	1989	84	Unavailable	-	6.0 (5) [2.0–13.3]
Algert (2001–03) [36]	Australia (2009)	2001	27	Unavailable	-	11.1 (3) [2.4–29.2]
**Heterogeneity I**^**2**^ **and P value**						**92.7%, p < 0.001**

^a^Data from Kalucy 1966–72, 1973–79, 1980–95 and 1986–90 were extracted from Kalucy (1994) [23]. Data from Laishram 1967–74 and 1986–90 were from Laishram (1993) [31].

**Fig 2:**
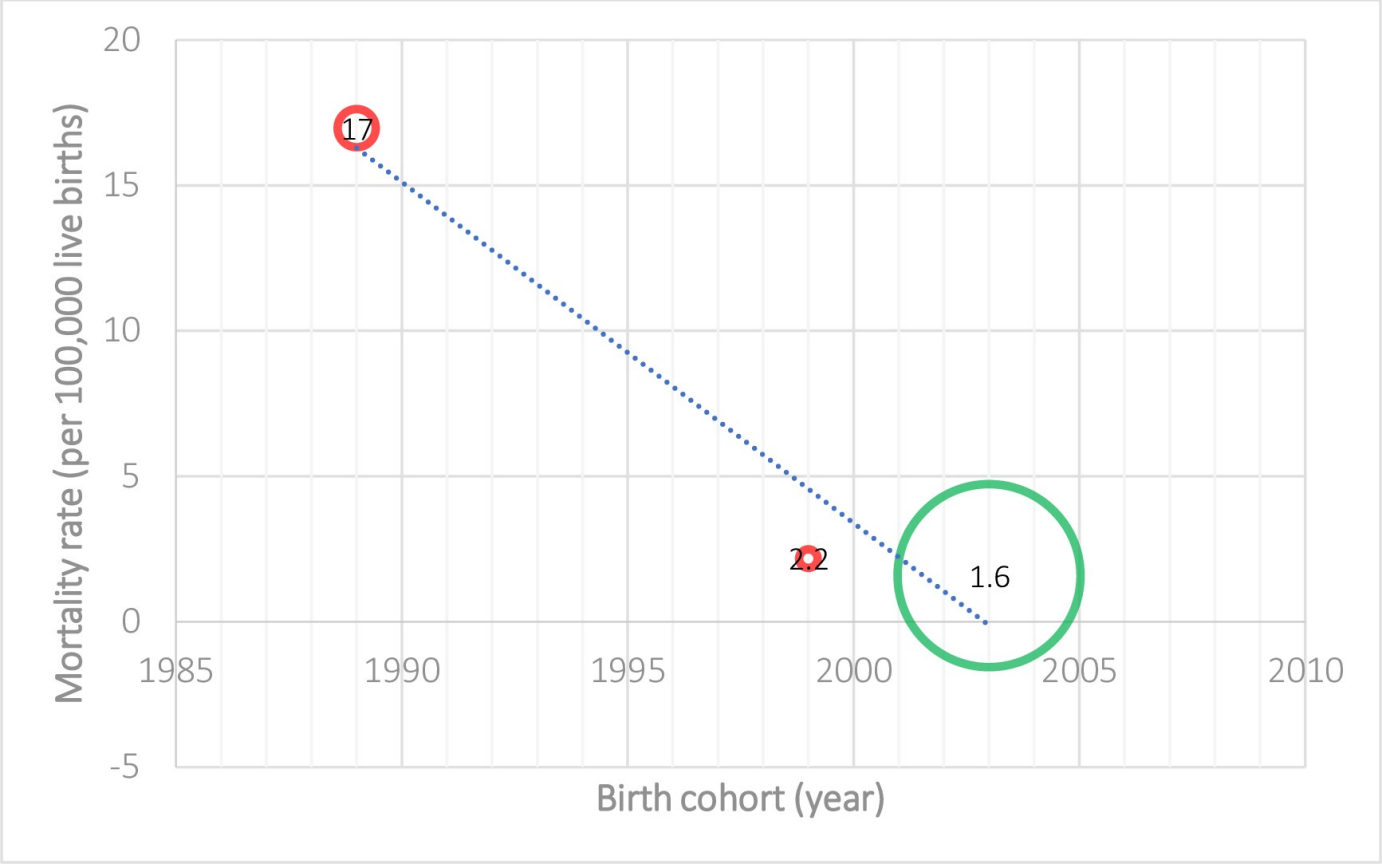
Neonatal mortality from spina bifida in Australia, the UK and the U.S. (Birth cohort: 1989–2003) [30,35,40].

**Fig 3:**
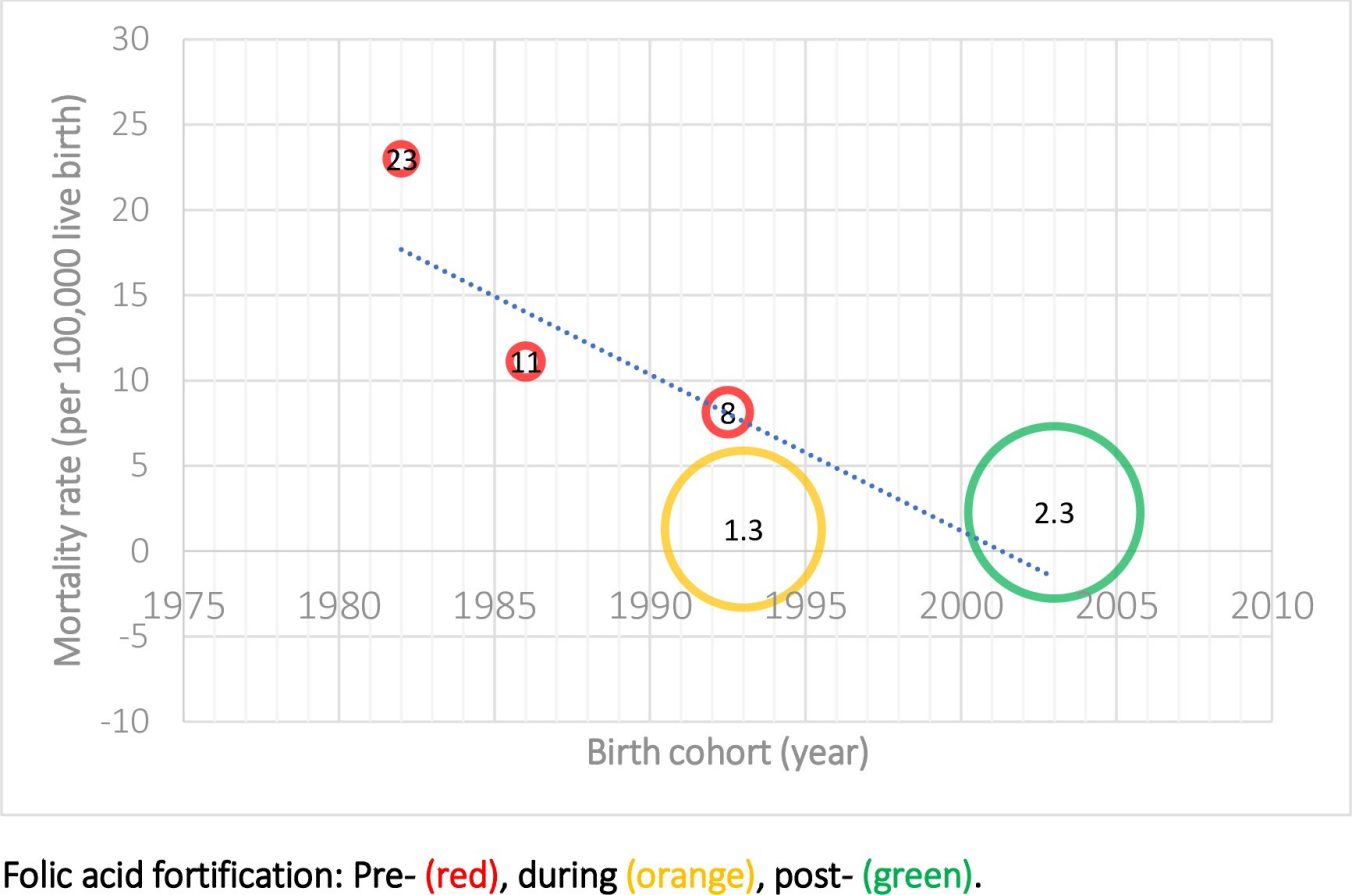
Infant mortality from spina bifida in the U.S. and Canada (Birth cohort: 1982–2003) [3,30,33,34].

**Fig 4:**
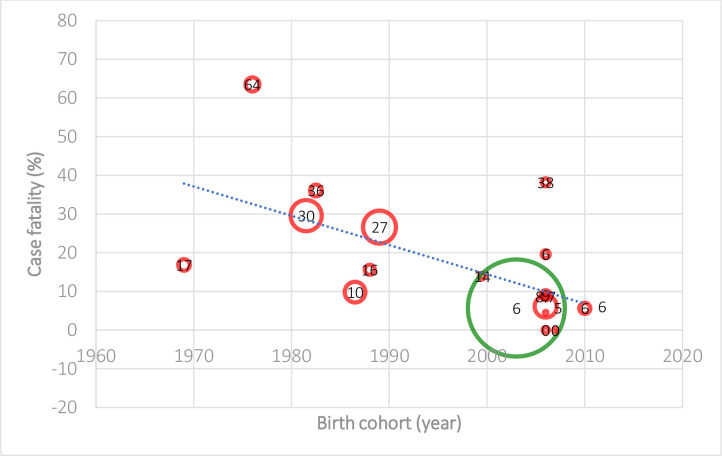
Neonatal case fatality from spina bifida in Australia, the U.S. and various European countries (Birth cohort: 1969–2010) [10,23,30,35,37–40,42,43].

**Fig 5:**
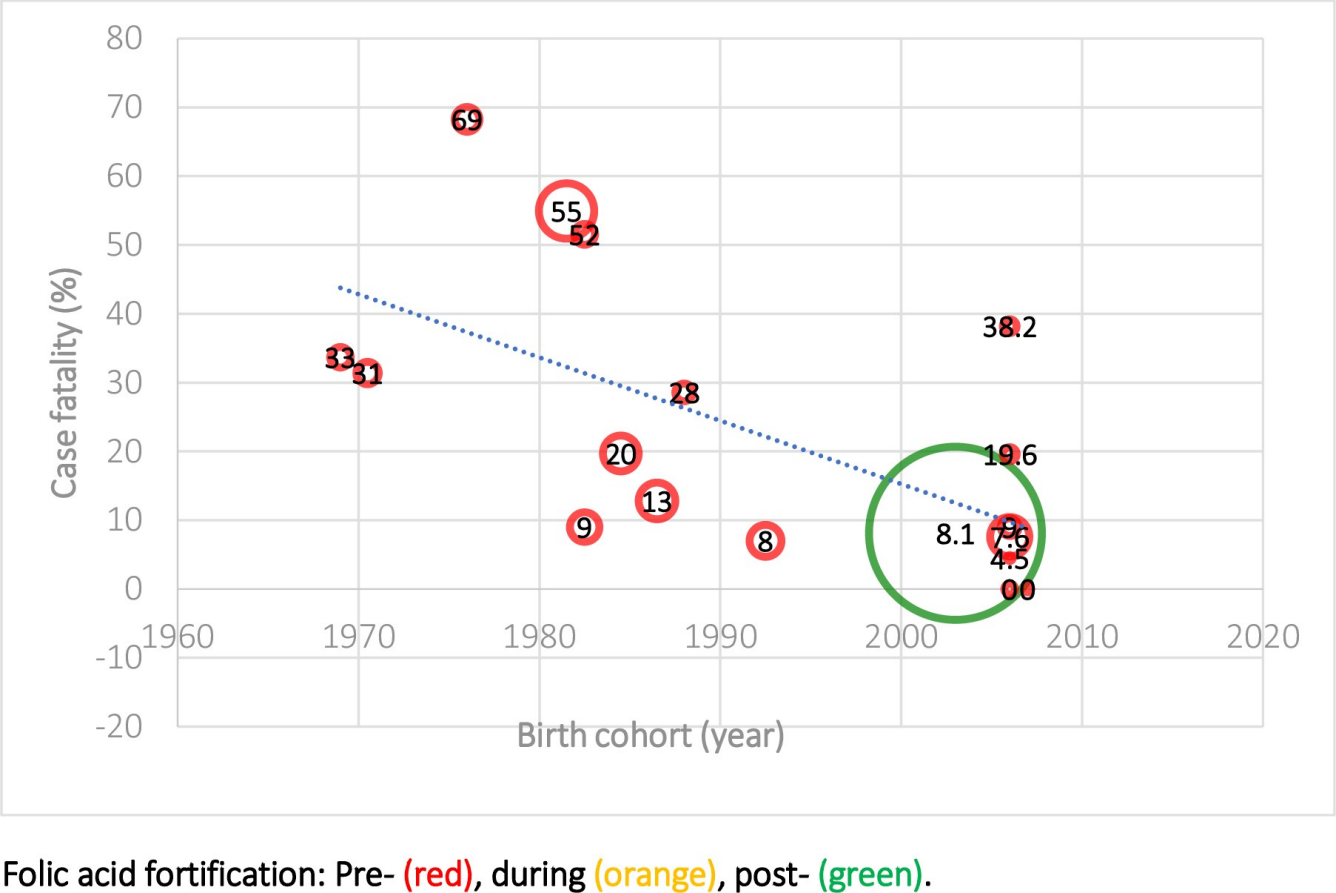
Infant case fatality from spina bifida in Australia, the U.S. and various European countries (1969–2003) [10,23,29–31,37,38,42,43].

There was considerable heterogeneity between studies for spina bifida NMR (I^2^ = 99.6%, p <0.001), IMR (I^2^ = 99.5%, p <0.001), neonatal case fatality (I^2^ = 98.7%, p < 0.001), and infant case fatality (I^2^ = 99.1%, p <0.001) (Tables 2 and 3). The fitted meta-regression shows a decrease over time in NMR [30,35,40] and IMR [3,30,33,34] due to spina bifida (slope = 7.14% and 4.76% decrease in death rate per 100, 000 live births per year respectively, p <0.001) (Figs 2 and 3), and in neonatal [10,23,30,35,37–40,42,43] and infant [29,10,23,30,31,37,38,42,43] case fatality (slope = 2.44% and 2.70% decrease per year respectively, p <0.001) (Figs 4 and 5). It is also important to note that the case fatality estimates from 1973–1979 were extremely high, which was due to strict selection criteria for surgical treatment of infants with spina bifida during the period [23]. In our sensitivity analysis, after the case fatality estimates from 1973–1979 reported by Kalucy et al. [23] were excluded, the decrease in neonatal and infant case fatality remained the same (slope = 2.44% and 2.70% decrease per year respectively, p <0.001).

### Risk factors for infant case fatality

**Fig 6:**
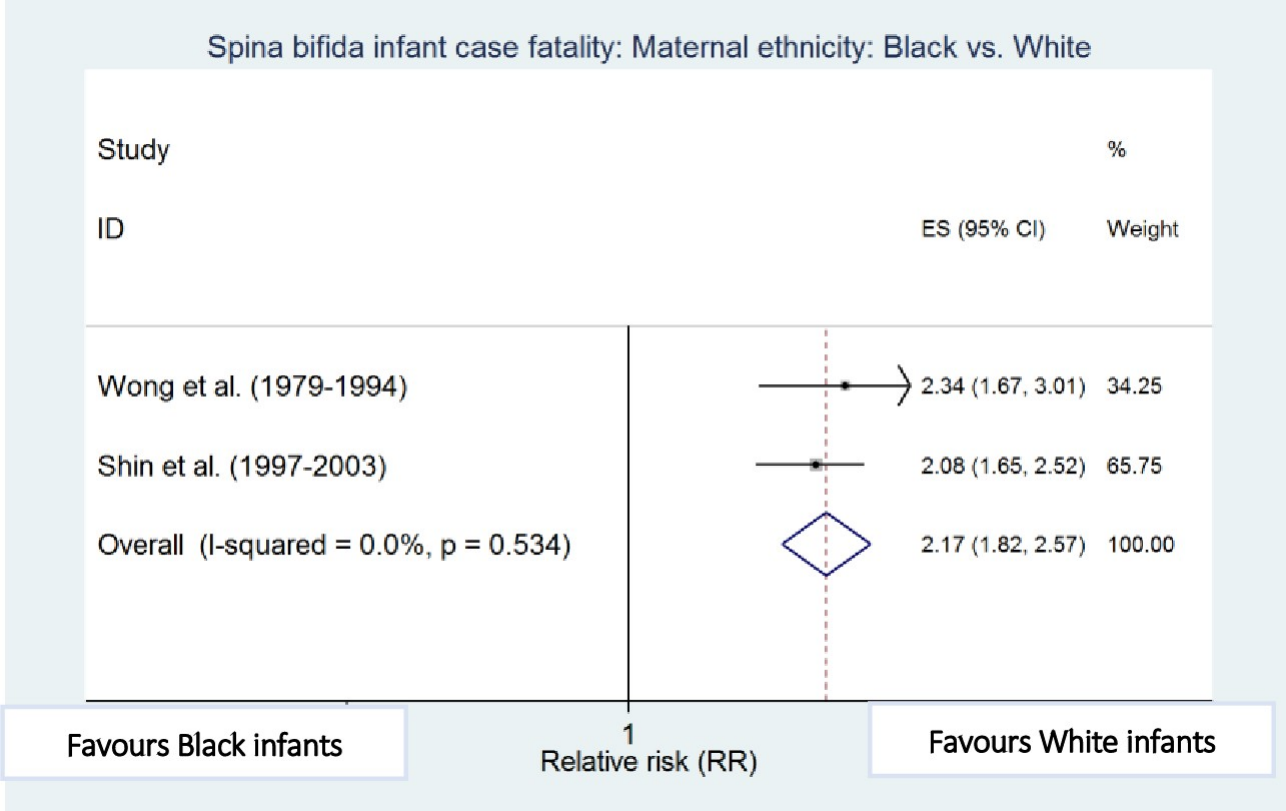
Maternal ethnicity. Spina bifida infant case fatality risk factors.

**Fig 7:**
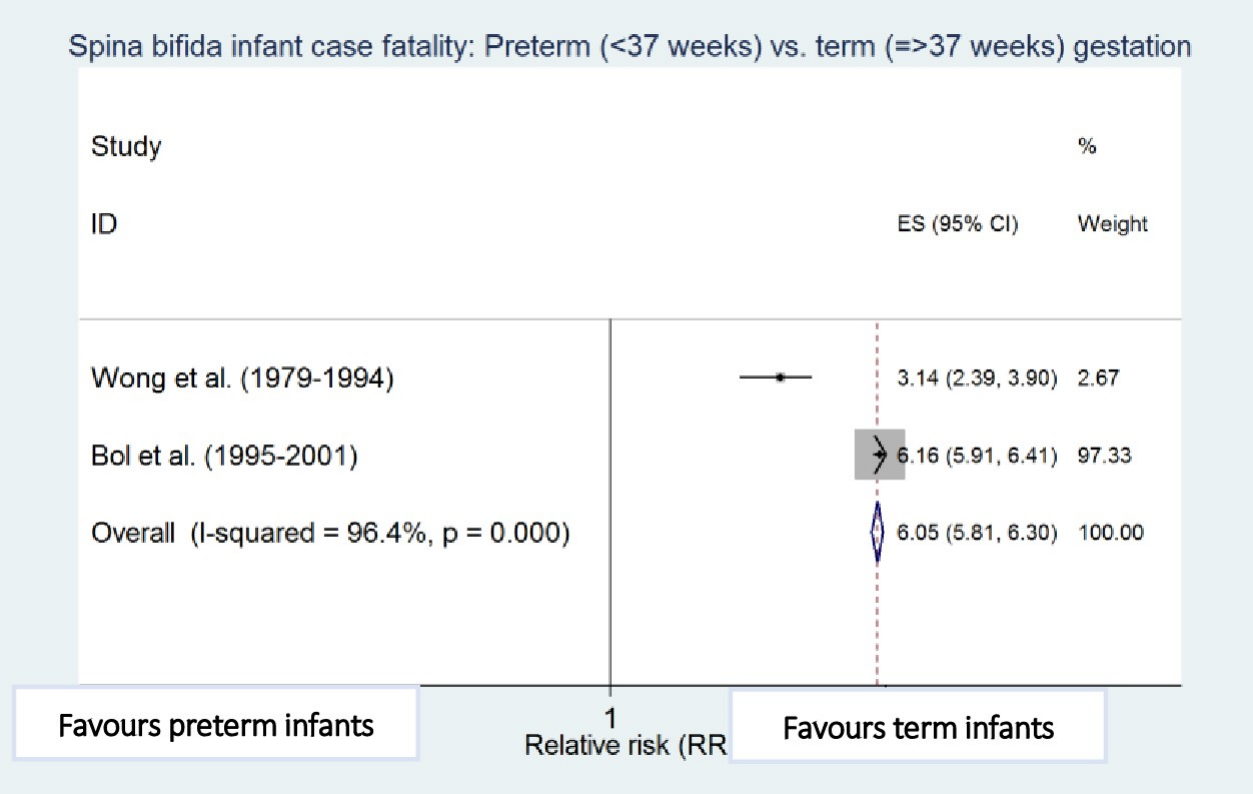
Gestational age. Spina bifida infant case fatality risk factors.

**Fig 8:**
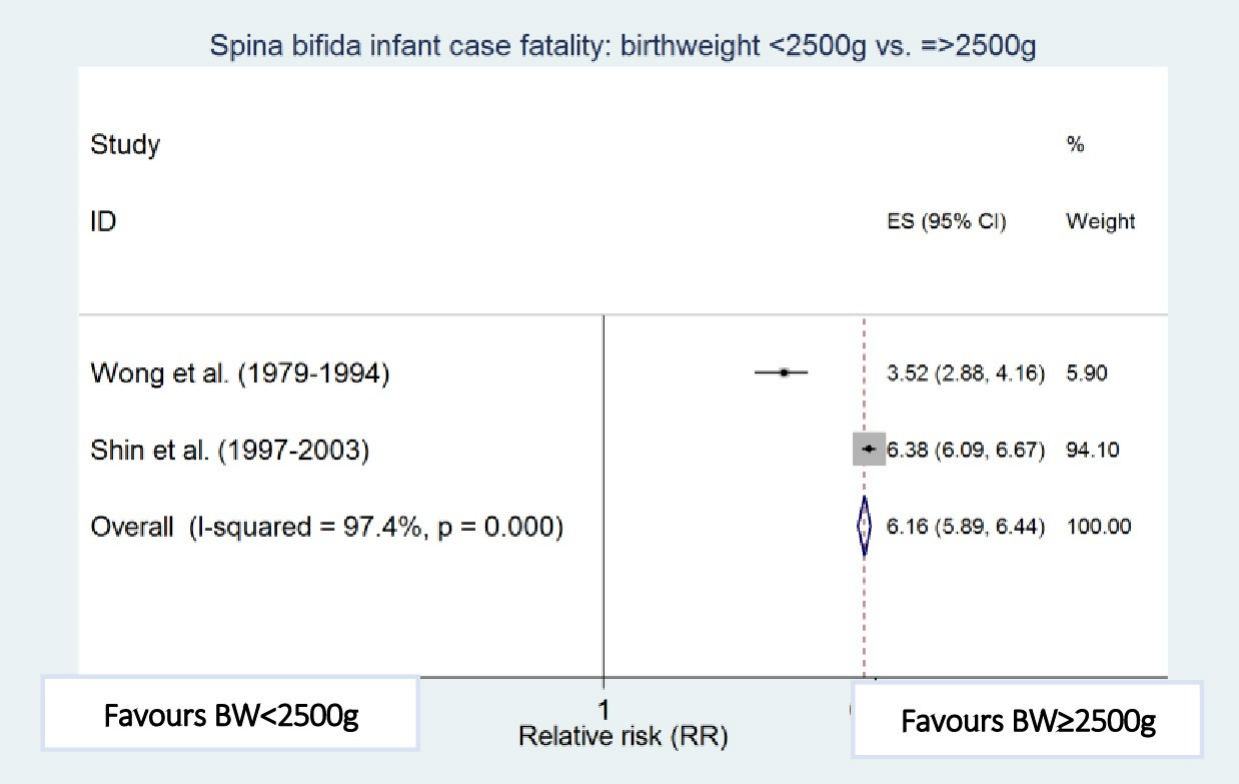
Birthweight. Spina bifida infant case fatality risk factors.

**Fig 9:**
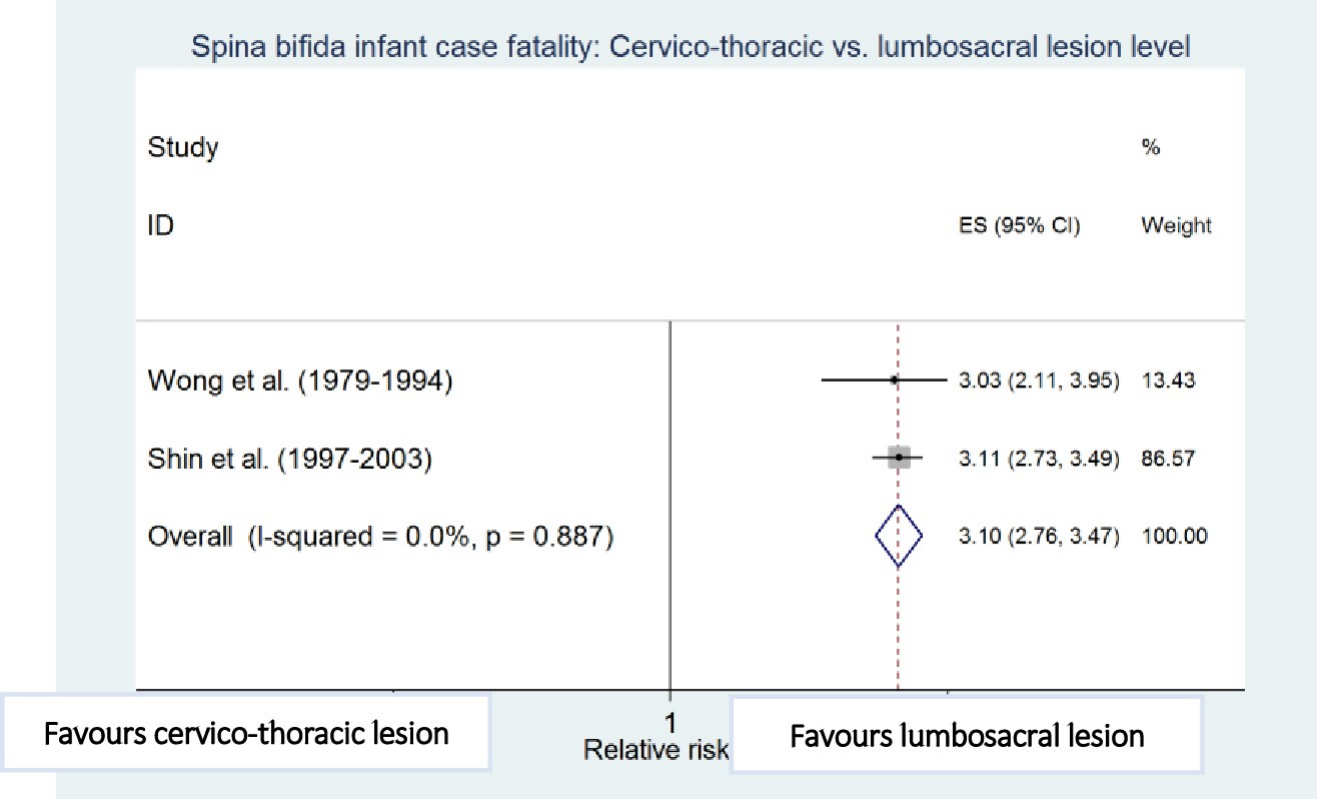
Lesion level. Spina bifida infant case fatality risk factors.

**Fig 10:**
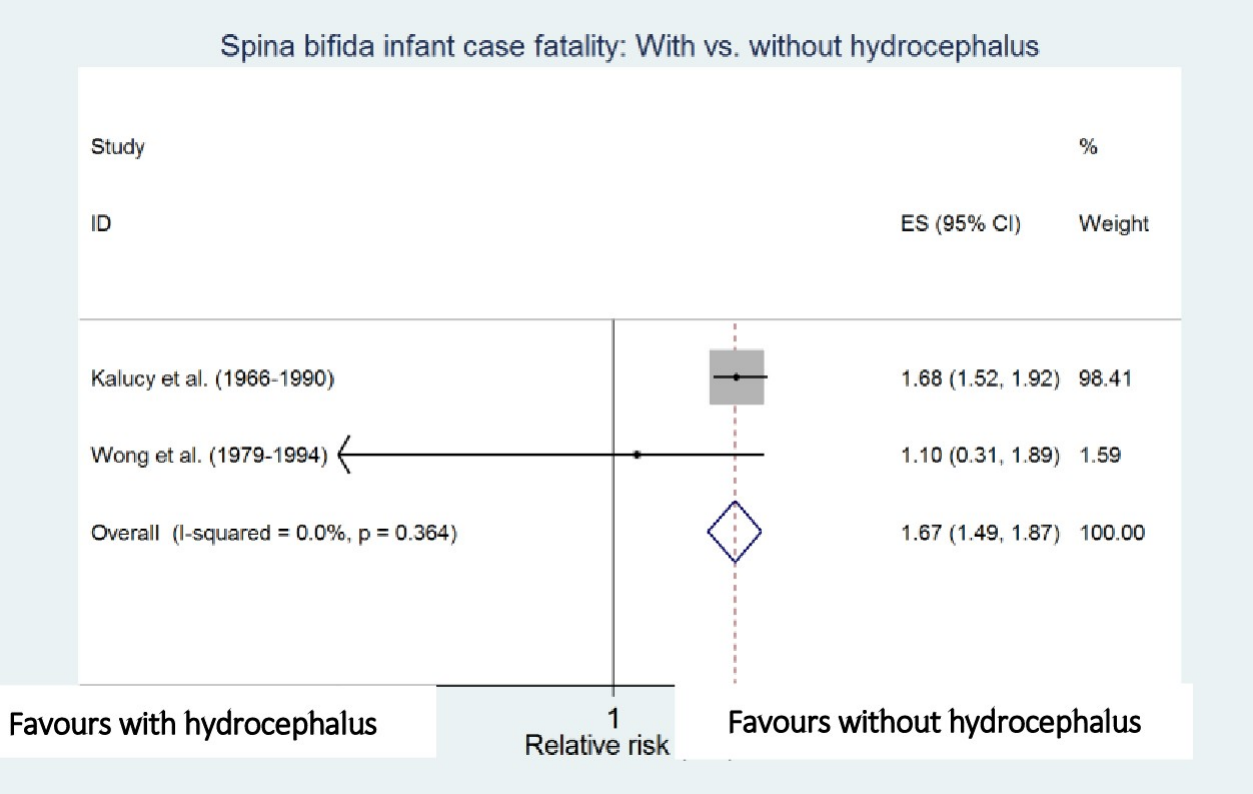
Presence of hydrocephalus. Spina bifida infant case fatality risk factors.

**Fig 11:**
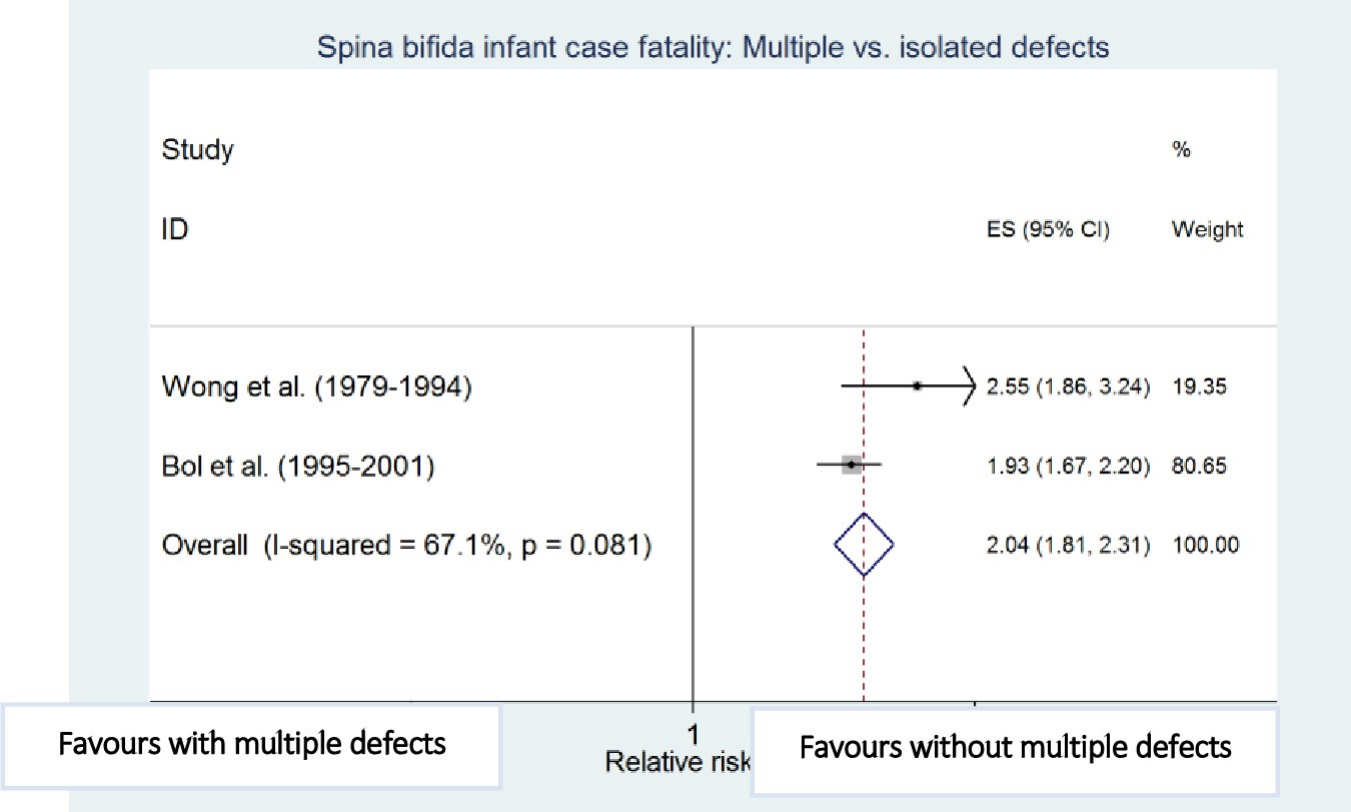
Presence of multiple anomalies. Spina bifida infant case fatality risk factors.

Meta-analyses showed that preterm birth (i.e. <37 weeks of gestation) [10,11], low birthweight (i.e. <2500g) [10,12], cervico-thoracic lesion level [10,12], presence of hydrocephalus [10,23], multiple defects [10,11], and maternal black ethnicity [10,12] were independently and significantly associated with an increased risk of death among infants with spina bifida after adjustment for other factors in the respective studies, with preterm birth (RR 4.45, 95% CI: 2.30–8.60) and low birthweight (RR 4.77; 95% CI: 2.67–8.55) being the strongest risk factors associated with increased spina bifida infant case fatality (Table 4 and Figs 6–11).

**Table 4:** Risk factors for spina bifida infant case fatality (relative risks estimates).

Risk factors	Studies (year of birth cohort)	Sample size	Case fatality relative risk (95% CI)
**Spina bifida Infant case fatality**			
**Black vs. White Ethnicity**	Wong (1979–1994) [10]	228	2.34 (1.67–3.01)
	Shin (1997–2003) [12]	1,315	2.08 (1.65–2.52)
Pooled estimate (95% CI) by random effects analysis			2.16 (1.82–2.57)
Pooled estimate (95% CI) by fixed effects analysis			*2*.*16 (1*.*82–2*.*57)*
*Heterogeneity I*^*2*^ *and P value*			*0*.*0%*, *p = 0*.*534*
**Male vs. Female**	Wong (1979–1994) [10]	234	1.07 (0.39–1.75)
	Shin (1997–2003) [11]	2,258	0.75 (0.44–1.05)
Pooled estimate (95% CI) by random effects analysis			0.81 (0.56–1.18)
Pooled estimate (95% CI) by fixed effects analysis			*0*.*81 (0*.*56–1*.*18)*
*Heterogeneity I*^*2*^ *and P value*			*0*.*0%*, *p = 0*.*419*
**Preterm vs. Term Gestation**	Wong (1979–1994) [10]	235	3.14 (2.39–3.90)
	Bol (1995–2001) [11]	2,761	6.16 (5.91–6.41)
Pooled estimate (95% CI) by random effects analysis			4.45 (2.30–8.60)
Pooled estimate (95% CI) by fixed effects analysis			*6*.*05 (5*.*81–6*.*30)*
*Heterogeneity I*^*2*^ *and P value*			*96*.*4%*, *p < 0*.*001*
**Birthweight <2500g vs. ≥2500g**	Wong (1979–1994) [10]	235	3.52 (2.88–4.16)
	Shin (1997–2003) [11]	2,255	6.38 (6.09–6.67)
Pooled estimate (95% CI) by random effects analysis			4.77 (2.67–8.55)
Pooled estimate (95% CI) by fixed effects analysis			*6*.*16 (5*.*89–6*.*44)*
*Heterogeneity I*^*2*^ *and P value*			*97*.*4%*, *p < 0*.*001*
**Cervico-thoracic vs. Lumbosacral level**	Wong (1979–1994) [10]	173	3.03 (2.11–3.95)
	Shin (1997–2003) [12]	1,674	3.11 (2.73–3.49)
Pooled estimate (95% CI) by random effects analysis			3.10 (2.76–3.47)
Pooled estimate (95% CI) by fixed effects analysis			*3*.*10 (2*.*76–3*.*47)*
*Heterogeneity I*^*2*^ *and P value*			*0*.*0%*, *p = 0*.*887*
**Hydrocephalus: Present vs. absent**	Kalucy (1966–1990) [23]	-	1.68 (1.52–1.92)
	Wong (1979–1994) [10]	219	1.10 (0.31–1.89)
Pooled estimate (95% CI) by random effects analysis			1.67 (1.49–1.87)
Pooled estimate (95% CI) by fixed effects analysis			*1*.*67 (1*.*49–1*.*87)*
*Heterogeneity I*^*2*^ *and P value*			*0*.*0%*, *p = 0*.*364*
**Multiple defects: Present vs. absent**	Wong (1979–1994) [10]	235	2.55 (1.86–3.24)
	Bol (1995–2001) [11]	2,841	1.93 (1.67–2.20)
Pooled estimate (95% CI) by random effects analysis			2.16 (1.66–2.81)
Pooled estimate (95% CI) by fixed effects analysis			*2*.*04 (1*.*81–2*.*31)*
*Heterogeneity I*^*2*^ *and P value*			*67*.*1%*, *p = 0*.*081*

Note: Pooled estimate Relative Risk (RR) was calculated by using random effects analysis. However if there were ≤3 studies, pooled estimate RR was also calculated using fixed effects analysis for comparison, with these results shown in *italics*.

Other significant risk factors for spina bifida case fatality reported in single studies included plurality (multiple vs. singleton: RR 2.57; 2.07–3.07) [12], induction of labour (yes vs. no: RR 2.49; 2.21–2.76) [11], maternal marital status (not married vs. married: RR 1.31; 1.04–1.57) [11], prenatal care (inadequate vs. adequate: RR 1.74; 1.36–2.12) [11]. Importantly, mandatory folic acid food fortification was significantly associated with a reduced hazard ratio of spina bifida infant case fatality (HR 0.68; 95% CI: 0.50–0.91) [11]. Infant sex, maternal age, maternal education, maternal smoking, previous live births and method of delivery were not significantly associated with case fatality for infants born with spina bifida [11,12]. (S2 Table).

### Quality appraisal

**Fig 12:**
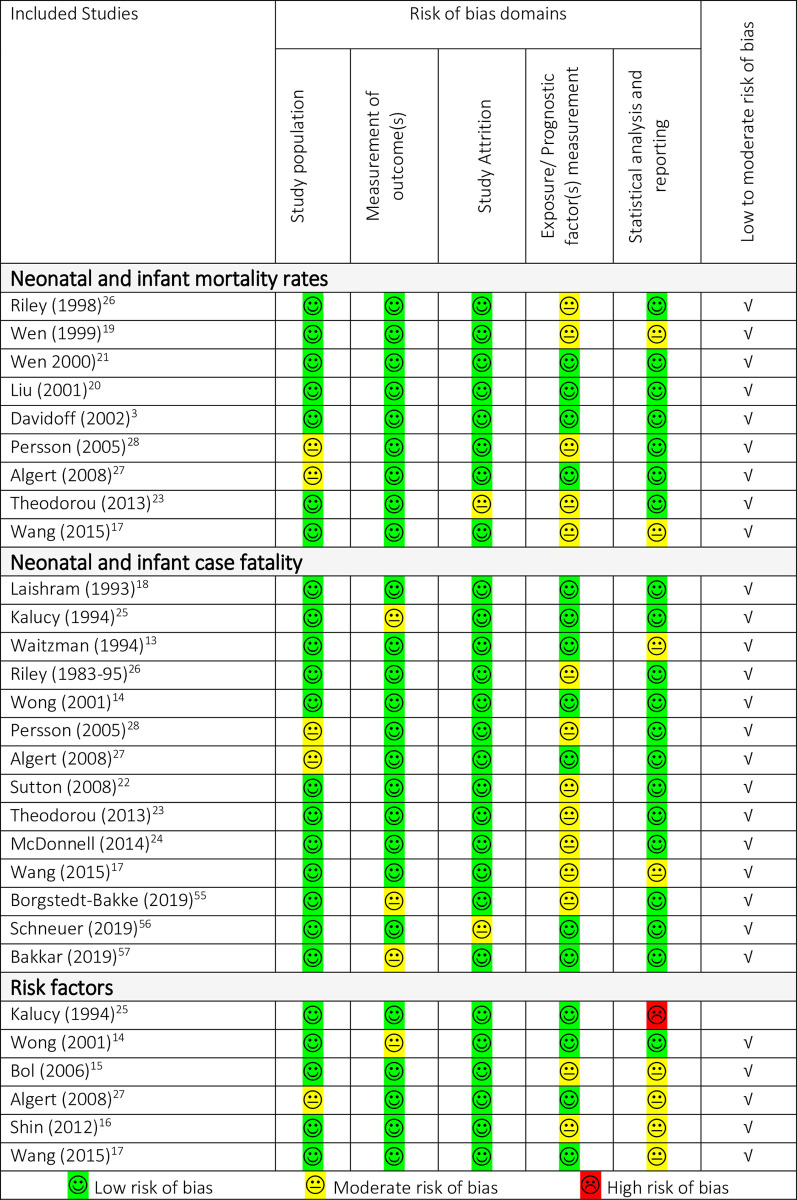
Risk of bias assessment summary table of the included studies.

Results of the quality appraisal of evidence are summarised in Fig 12. The majority of the included studies satisfied the study population domain as they were national or register-based. The exposure/ prognostic factors measurement domain was satisfied in about half of the included studies, this was mainly due to the differences in treatment and preventative interventions available over time and between countries of the studies, as well as variations between studies in reporting syndromal and multiple defects which is a major prognostic factor. The statistical analysis and reporting domain was satisfied only in one-fifth of the included studies due to a combination of concurrent cases [11,38], missing data, unreported 95% CI [12,29], and the potential for incomplete ascertainment of deaths particularly in early surveillance programmes [11,30,35,41]. In addition, some studies did not perform mortality analysis but only reported the number of infants alive or dead, in which cases censoring was not accounted for. Infant mortality and case fatality estimates may have been underestimated in these studies.

## Discussion

This systematic review included 20 population-based cohort studies with a total study population of over 30 million liveborn infants, in which approximately 12,000 liveborn infants were affected by spina bifida; outcomes were reported based on generally high-quality evidence from studies mainly with a low to moderate risk of bias. The review spanned 49 years with births from 1966 to 2015. Variation in study period is perhaps the main source of heterogeneity for the mortality estimates. This is largely indicative of different clinical and interventional exposures to which the patient populations were subject to over time.

Spina bifida infant mortality encompasses both to the chance of being born with spina bifida as well as surviving with the condition during infancy (i.e. case fatality), both of which have significantly decreased over time. There are several possible mechanisms including:

First, primary prevention: Five large population-based US cohort studies in this review have consistently shown a significant decline over time in spina bifida infant mortality post mandatory folic acid fortification of U.S. grain supply introduced in September 1998 [3,13,30,33–45]. This finding is consistent with long term surveillance data from the U.S., Canada, Chile and Costa Rica where folic acid food fortification had been successfully implemented, showing that the NTD birth prevalence can be reduced over time to as low as 5–6 per 100,000 pregnancies [9,46]. Indeed, the original large (n = 1,817 women at high risk of having a pregnancy with a NTD) international randomised double-blind prevention trial conducted by the MRC Vitamin Study Research Group showed a 72% protective effect against the occurrence of an NTD (i.e. spina bifida, encephalocele and anencephaly) pregnancy for women taking 4 mg folic acid supplementation daily prior to conception [5]. Similarly, in a large population-based cohort study, Berry et al. (1999) have shown that periconceptional intake of folic acid reduce the risk of NTDs in areas with variable prevalence rates in China [47]. These findings established the specific role of folic acid supplementation in the prevention of NTDs.

Second, the studies by Kalucy et al. (1994) [23], Riley et al. (1998) [35], Wen et al. (1999) [32] and Davidoff et al. (2002) [3] have shown that spina bifida infant mortality and case fatality declined over time prior to national implementation of folic acid food fortification in Australia and the U.S‥ This finding suggests that improved technology in and access to prenatal screening and diagnosis over time in high-income countries have led to significant declines in both late fetal death and birth of spina bifida affected infants as a result of early prenatal diagnosis and termination of pregnancy [23,48]. According to EUROCAT data, over 50–75% of cases of spina bifida are terminated in Europe [49].

Possible explanations for the decline in spina bifida infant case fatality over time include:

First, folic acid food fortification: This review has shown a significant decline over time in spina bifida infant case fatality post mandatory folic acid fortification of the U.S. grain supply [3,13,30,33,34]. Infants born with a spina bifida during the period of mandatory fortification were almost one-third less likely to die in infancy compared to infants born with a spina bifida during pre-fortification period [11]. Studies suggest that folic acid food fortification, may reduce the risk of severe types of spina bifida by moving the lesion caudally along the developing spine [11,50–52], as upper-level spina bifida is associated with more severe anomalous brain development and more system wide complications compared with lower-level spina bifida [42,53].

Second, advances in medical and surgical interventions: Studies have suggested that improved ventilator support in neonatal intensive care and the use of antibiotics to treat CNS infections have provided better treatment of spina bifida and associated co-morbidities such as prematurity and low birthweight which have been shown as the strongest predictors for infant deaths in this review [54]. In addition, advances in surgical treatment for spina bifida and associated hydrocephalus and cardiac defects may also have contributed to the reductions in case fatality [31,50,55–60].

Regarding other risk factors, mortality among Black and Hispanic infants with spina bifida has declined with time but remains consistently higher when compared to White infants [12]. Possible explanations include barriers to access to health care particularly among women of lower socioeconomic classes [12,61,62]. Hispanic populations also have a high incidence of C677T methylenetetrahydrofolate reductase (MTHFR) homozygous genotype mutations [62,63] which have been shown to be associated with upper level spina bifida defects [64]. Fetuses with an anomaly often have other developmental effects during gestation which may also lead to preterm birth [65,66]. Therefore, preterm birth and congenital anomalies are likely to have a multiplicative effect on infant mortality. Multiple anomalies are an indicator of disease complications and severity, and hence tend to be associated with poorer prognosis when major systems are involved, compared to isolated anomalies [43,67].

The main strengths of this review include its comprehensive search strategy with no language restriction, robust inclusion criteria and complete coverage of liveborn spina bifida population-based studies. Unpublished relevant data were obtained from study authors and included in the analysis where possible. A further strength is the generally high quality of the included studies with the majority of studies rated as low to moderate risk of bias according to the refined QUIP and Cochrane RoB tool [26,28].

There are several limitations. First, mortality in infants with spina bifida depends on various factors including health care systems, treatment, termination of pregnancy and ability to track and link all cases with death registers. Without the availability of these data, it is not possible to determine the true underlying cause of the reduction in infant case fatality and mortality rates. In addition, we could not verify whether multiple defects and syndromal spina bifida were included or excluded in the majority of the included studies. Consequently, these potentially caused high variability and heterogeneity in the estimates. Second, study data about risk factors for spina bifida case fatality were limited; in particular, data about women intake of folic acid supplement was not available in most of the included studies, which is an important factor to account for. Finally, cases of multiple defects and syndrome associated with spina bifida cannot be completely excluded from the analysis as they cannot be reliably identified in most of the included studies.

## Conclusions

This study has reviewed the evidence about spina bifida infant mortality in high-income countries. This study has shown that a decline in spina bifida associated infant/neonatal mortality and case fatality were consistently observed, in which advances in medical and surgical treatment, and mandatory folic acid food fortification likely to play an important role. Preterm birth and low birthweight are strongest risk factors associated with increased case fatality of infants with spina bifida, which warrant particular attention from clinicians caring for these vulnerable babies.

## Supporting information

S1 Checklist PRISMA 2009 checklist. (DOC)

S1 Table Detailed search terms using PubMed online database. (DOCX)

S2 Table Other risk factors for spina bifida infant case fatality from single studies (relative risk estimates). (DOCX)
