# Supplementary material for: Neonatal and infant mortality associated with spina bifida: A systematic review and meta-analysis
Source: PLoS One. 2021 May 12;16(5):e0250098. doi: 10.1371/journal.pone.0250098 (PMC8115829; doi:10.1371/journal.pone.0250098)
Supplement: S1 Table — (DOCX) [file pone.0250098.s002.docx]

**Infant mortality associated with spina bifida: A systematic review and meta-analysis (Ho et al.)**

**Additional information**

**Supplementary Table 1: Detailed Search Terms Using PubMed Online Database**

| (((neural tube defect* OR congenital brain anomal* OR congenital brain malformation* OR congenital brain abnormal* OR congenital spinal anomal* OR congenital spinal malformation* OR congenital spinal abnormal* OR congenital central nervous system anomal* OR congenital central nervous system malformation* OR congenital central nervous system abnormal* OR spina bifida* OR myelomeningocele* OR meningomyelocele* OR meningocele* OR encephalocele*[MeSH Terms])) AND (newborn* OR neonat* OR infan* OR perinatal*[MeSH Terms])) AND (mortality OR death* OR survival*[MeSH Terms]) Filters: Publication date from 1990/01/01 to 2020/08/31; Humans |
| --- |
