# Supplementary material for: Neonatal and infant mortality associated with spina bifida: A systematic review and meta-analysis
Source: PLoS One. 2021 May 12;16(5):e0250098. doi: 10.1371/journal.pone.0250098 (PMC8115829; doi:10.1371/journal.pone.0250098)
Supplement: S2 Table — (DOCX) [file pone.0250098.s003.docx]

**Supplementary Table 2: Other risk factors for spina bifida infant case fatality from single studies (relative risk estimates)**

| **Risk factors in single studies** | **Studies (year of birth cohort)** | **Sample size** | **Case fatality relative risk (95% CI)** |
| --- | --- | --- | --- |
| Maternal and other socio-demographical factors | | | |
| Maternal age: ≥35 years vs. <35 years | Shin (1997-2003)^16^ | 2,258 | 1.05 (0.61-1.49) |
| Maternal education: high school or less vs. more | Bol (1995-2001)^15^ | 2,743 | 1.10 (0.83-1.36) |
| Maternal smoking: Yes vs. No | Bol (1995-2001)^15^ | 2,752 | 0.90 (0.53-1.27) |
| Maternal marital status: Not married vs. married | Bol (1995-2001)^15^ | 2,274 | 1.31 (1.04-1.57) |
| Maternal ethnicity: Hispanic vs. White | Shin (1997-2003)^16^ | 1,986 | 1.33 (1.00-1.65) |
| Previous live births: Yes vs. No | Bol (1995-2001)^15^ | 2,769 | 1.17 (0.92-1.42) |
| Prenatal care: Inadequate vs. Adequate | Bol (1995-2001)^15^ | 1,267 | 1.74 (1.36-2.12) |
| Infant and clinical factors | | | |
| Male vs. Female | Bol (1995-2001)^15^ | 2,701 | 0.93 (0.69-1.17) |
| Multiple vs. Singleton | Shin (1997-2003)^16^ | 2,252 | 2.57 (2.07-3.07) |
| Induction of labour: Yes vs. No | Bol (1995-2001)^15^ | 2,775 | 2.49 (2.21-2.76) |
| Interventional factors | | | |
| Mandatory folic acid food fortification vs. prefortification | Bol (1995-2001)^15^ | 2,139 | 0.81 (0.54-1.09) |
| Method of delivery: Caesarean vs. Vaginal | Bol (1995-2001)^15^ | 2,655 | 0.50 (0.25-0.76) |
| Type of hospital: Delivered at co-located paediatric surgical units vs. others | Algert (2001-2003)^27^ | 27 | 0.29 (0.00-2.56) |
